# Effects of spent substrate of oyster mushroom (*Pleurotus ostreatus*) on ruminal fermentation, microbial community and growth performance in Hu sheep

**DOI:** 10.3389/fmicb.2024.1425218

**Published:** 2024-10-23

**Authors:** Mu-Long Lu, Guo-Hong Yuan, Halidai Rehemujiang, Chang-Chang Li, Li-Hong Hu, Ping-Ping Duan, Li-Dong Zhang, Qi-Yu Diao, Kai-Dong Deng, Gui-Shan Xu

**Affiliations:** ^1^College of Animal Science and Technology, Tarim University, Alar, China; ^2^Key Laboratory of Livestock and Forage Resources Utilization around Tarim, Ministry of Agriculture and Rural Affairs, Tarim University, Alar, China; ^3^Institute of Feed Research/Key Laboratory of Feed Biotechnology of the Ministry of Agriculture and Rural Affairs, Chinese Academy of Agricultural Sciences, Beijing, China; ^4^College of Animal Science and Food Engineering, Jinling Institute of Technology, Nanjing, Jiangsu, China

**Keywords:** white-rot fungi, fungal pretreatment, gossypol, rumen microorganisms, spent mushroom substrate

## Abstract

**Introduction:**

The study aimed to evaluate the effects of Pleurotus Spent Mushroom Substrate (P.SMS) on the rumen microbiota, encompassing bacteria and fungi, as well as their interactions in Hu sheep.

**Methods:**

A total of forty-five 3-month-old Hu sheep were randomly assigned to five groups. Each group was fed diets in which whole-plant corn silage (WPCS) was substituted with P.SMS at varying levels: 0% (CON), 5% (PSMS5), 10% (PSMS10), 15% (PSMS15), or 20% (PSMS20).

**Results:**

The results indicated that higher proportions of P.SMS during the experimental period might have a detrimental effect on feed utilization efficiency, kidney function, and blood oxygen-carrying capacity. Notably, moderate levels of P.SMS, specifically below 15%, were associated with improvements in rumen NH_3_-N levels and absorption capacity. The results indicated that (1) PSMS20 exhibited a significantly higher feed-to-gain ratio compared to CON (*P* < 0.05); (2) PSMS15 showed a significantly higher NH_3_-N content than CON, PSMS5, and PSMS20. Additionally, PSMS10 and PSMS20 had elevated concentrations of NH_3_-N compared to CON and PSMS5 (*P* < 0.05); (3) The length and width of rumen papillae were significantly greater in PSMS20 compared to CON and PSMS5 (*P* < 0.05); (4) Creatinine levels were significantly higher in PSMS20 than in CON, PSMS5, and PSMS10 (*P* < 0.05); (5) By the conclusion of the experiment, hemoglobin concentration in PSMS20 showed a significant increase compared to CON (*P* < 0.05). Furthermore, the addition of P.SMS influenced microorganisms at both the phylum and genus levels: (1) At the phylum level, the prevalence of *Patescibacteria* was significantly lower in PSMS20 compared to the other groups; (2) PSMS15 exhibited significantly higher relative abundances of *Basidiomycota* compared to CON and PSMS10, while PSMS20 also demonstrated significantly higher relative abundances compared to CON (*P* < 0.05); (3) At the genus level, the prevalence of *Candidatus_Saccharimonas* in PSMS20 was significantly lower than in PSMS5, PSMS10, and PSMS15. Conversely, the prevalence of *Phanerochaete* in PSMS15 was notably higher than in CON and PSMS10, and it was also significantly elevated in PSMS20 compared to CON (*P* < 0.05); (4) Correlation analysis indicated no significant correlation between changes in the structure of bacterial and fungal communities.

**Discussion:**

Considering these findings, a high percentage of P.SMS negatively impacted feed utilization efficiency, blood oxygen carrying capacity, and kidney function, while a moderate percentage of P.SMS promotes rumen absorption capacity, indicating that feeding 10% P.SMS is optimal.

## 1 Introduction

Mushrooms have traditionally been utilized in Asian countries, particularly in China, for their medicinal properties and as functional foods due to their high-quality proteins, carbohydrates, minerals, vitamins, and low-fat content (Martin et al., 2023). Spent mushroom substrate (SMS) typically consists of the plant biomass that is discarded after mushroom harvesting cycles (Lucas De Jesus et al., 2023). With the growing demand for mushrooms, approximately five times the amount of SMS is generated with each kilogram of mushrooms produced, resulting in significant waste. A previous study highlighted the nutraceutical potential of SMS, particularly for its prebiotic properties (Baptista et al., 2023). A review noted that the mycelium and fruiting bodies exhibit different compositions based on their environments, but this does not compromise their safety or quality when cultivated under controlled conditions (Berger et al., 2022). While research on the feeding of mycelium remains limited, existing studies suggest that mycelium consumption can enhance the body’s immunity and possess anti-inflammatory properties (Shin et al., 2021; Yang et al., 2022). Nonetheless, the majority of SMS has been either incinerated or discarded as waste, leading to the loss of valuable biological resources and contributing to severe environmental pollution (Huang et al., 2023). Therefore, the full utilization of SMS shows promise, but investigation is needed to explore the impact of SMS feed utilization on ruminant animals.

Pleurotus mushrooms are recognized for their safety and edibility, along with diverse biotechnological and environmental applications. This versatility is largely attributed to their non-specific oxidative enzymatic systems and ligninolytic peroxidases, which play a crucial role in various biodegradation processes, including the fungal pretreatment of lignocellulosic biomass for sustainable waste management (Kainthola et al., 2021; Scholtmeijer et al., 2023). *Pleurotus* spp. exhibit an exceptional ability to selectively mineralize lignin through the production of various extracellular oxidizing enzymes, thereby establishing an effective lignin degradation system (Knop et al., 2015; van Kuijk et al., 2015). This process enhances the accessibility of cellulose and hemicellulose for the rumen microbe. A recent study by Agustinho et al. (2021) identified three lignin-degrading enzymes—lignin peroxidase, and manganese peroxidase—in the spent mushroom substrate of *Pleurotus ostreatus* (P.SMS). When incorporated into whole corn plant ensiling fermentation, these enzymes contributed to a reduction in lignin concentration. Additionally, research by Xu et al. (2010) indicated that increasing the concentration of SMS in total mixed ration silage tended to elevate the acetate to propionate ratio and pH in the rumen. Song et al. (2007) demonstrated that feeding finishing Berkshire pigs with fermented oyster mushroom by-products influenced multiple indicators during the fattening process. Furthermore, a study conducted by Huang et al. (2023) revealed that the addition of fermented SMS significantly improved the diversity and abundance of the rumen bacterial community. Our previous study pointed out that the inclusion of a high proportion (20%) of P.SMS exhibited a favorable feeding effect during the initial 2 months of the fattening period; however, as the feeding duration was extended, the effectiveness of the feeding began to decline (Guohong et al., 2023). Despite these findings, there remains a notable lack of studies directly investigating the effects of feeding SMS to animals, as well as limited research on its impact on rumen fungi.

The microbial composition of an animal’s digestive tract plays a crucial role in various physiological responses of the organism. The complex relationship between the host and its gut microbiota influences various aspects, including nutrient digestion and absorption, immune system modulation, overall health, and even behaviors (de Vos et al., 2022; Lamontagne et al., 2023; Ma Y. et al., 2022; Martinez-Guryn et al., 2018; Zhang X. et al., 2022). The degradation of plant cell wall polysaccharides in the rumen is known to be associated with ruminal bacteria, protozoa, and fungi (Rehemujiang et al., 2023).

Given that SMS incorporates mycelium, a unique type of fungus, and that Pleurotus-treated substrates enhance microbial access to cellulose and hemicellulose, we hypothesized that the addition of P.SMS would create additional utilization sites for rumen microbes or alter the rumen flora in sheep. Our objective was to investigate the impact of direct SMS feeding on Hu sheep by examining growth performance, rumen, and blood indices, as well as the rumen microbial community. This study focused on assessing the effects of incorporating SMS at various supplementation levels, replacing whole-plant corn silage (WPCS), on the microbial community structure in the rumen of sheep. We anticipated that this research would provide a novel perspective on SMS feeding, offering valuable guidance and insights for related research and practical applications.

## 2 Materials and methods

### 2.1 Ethics committee approval

The animal study was reviewed and approved by the animal ethics committee of Tarim University (2,024,049 and 2,024,067), written informed consent was obtained from the owners for the participation of their animals in this study.

### 2.2 Spent mushroom substrate from *Pleurotus ostreatus*

P.SMS was cultivated using a substrate consisting of cultured from 70% cottonseed hulls, 20% sawdust, 7% wheat bran, and 3% lime sourced from a mushroom plantation located on the outskirts of Alar City, Xinjiang. The chemical composition of P.SMS and WPCS used in the experiment is described in Table 1.

**Table 1:** The chemical composition^*^ of P.SMS and WPCS used in the experiment.

Nutrient levels^†^	P.SMS Content (%)	WPCS Content (%)
Crude protein	8.70	6.77
Crude fat	2.41	4.40
Crude ash	29.41	13.39
Neutral detergent fibers	43.97	59.97
Acid detergent fibers	18.69	37.08
Calcium	0.63	0.59
Phosphorus	0.04	0.23

* The chemical composition was based on dry matter basis.

† Nutrient levels were obtained from chemical analysis.

### 2.3 Experimental animals and group design

A completely randomized experimental design was employed in this study. Forty-five 3-month-old male Hu sheep with an average weight of 30.0 ± 1.2 kg were selected and randomly assigned to 5 groups. These groups received varying levels of spent mushroom substrate (SMS) as treatments: CON (0%), PSMS5 (5%), PSMS10 (10%), PSMS15 (15%), and PSMS20 (20%). Each treatment group consisted of 9 sheep. The basic diet, formulated to meet the requirements of a 30-kg sheep with a daily gain of 200 g, is detailed in Table 2. The diet was administered twice daily, once in the morning at 10:00 and again at 19:00. An adaptation period of 10 days preceded a 90-day trial period. The feed ingredients in the basal diet were sourced from the farm, and their percentages were adjusted to meet the energy requirements for growth. The formulation remained unchanged throughout the feeding process; only the quantity fed was adjusted based on leftovers. During the trial, all sheep were housed individually in a partially open enclosure, provided with free access to salt blocks and clean water, and exposed to natural light and a cool environmental temperature.

**Table 2:** Test diet composition and nutrition level (dry matter basis) %.

Items	Groups^⸸^				
	CON	PSMS5	PSMS10	PSMS15	PSMS20
Ingredients					
P.SMS	0.00	5.00	10.00	15.00	20.00
Whole Corn silage	52.36	47.36	42.36	37.36	32.36
Cornstalk	7.64	7.64	7.64	7.64	7.64
Corn	12.08	12.08	12.08	12.08	12.08
Wheat bran	6.32	6.32	6.32	6.32	6.32
Soybean meal	19.10	19.10	19.10	19.10	19.10
NaCl	1.00	1.00	1.00	1.00	1.00
Premix^*^	1.50	1.50	1.50	1.50	1.50
Total	100.00	100.00	100.00	100.00	100.00
Nutrient levels^†^					
ME (MJ/kg)^‡^	9.10	9.18	9.27	9.36	9.44
CP	14.41	14.51	14.60	14.69	14.81
NDF	42.53	41.73	40.93	40.13	39.33
ADF	25.51	24.56	23.62	22.67	21.73
Ca	0.49	0.62	0.75	0.86	1.02
TP	0.38	0.38	0.37	0.37	0.36

* Premix provided the following per kg in basic diets: 10 mg of iron, 135 mg of manganese, 100 mg of zinc, 0.5 mg of cobalt, 12.5 mg of copper, 0.3 mg of selenium, 1.5 mg of iodine, 1,400 IU of vitamin A, 500 IU of vitamin D, and 50 mg of vitamin E.

† ME, metabolic energy; CP, crude protein; NDF, neutral detergent fiber; ADF, acid detergent fiber; TP, total phosphorus.

‡ The metabolic energy and metabolizable protein in the nutritional level were calculated reference to the Nutritional Requirements of Sheep for Meat in China, and the rest were measured values.

⸸ P.SMS replaced WPCS at levels of 0%, CON; 5%, PSMS5; 10%, PSMS10; 15%, PSMS15; or 20%, PSMS20.

### 2.4 Sample collection and processing

#### 2.4.1 Feed intake and growth performance

The feed intake was monitored daily, with leftovers from the previous day collected before the morning feed, dried, and weighed to determine the dry matter intake (DMI) and feed-to-gain ratio (F/G). Sheep were weighed before morning feeding on the 1st and 90th days to compute the average daily weight gain (ADG) over the feeding period.

#### 2.4.2 Rumen parameters

One day prior to slaughter, a veterinarian examined the sheep to confirm the absence of gastrointestinal diseases. At the conclusion of the experiment, 25 sheep were randomly chosen from each group (5 sheep per group) and subjected to a 24-h fast, and 12 h without water before slaughter. All sheep were slaughtered, and their rumen was isolated. All the rumen fluid from each sheep was collected by filtering the digest contents through sterile 4-layer gauze. We then froze and stored 150 mL of rumen fluid from each sheep. The pH of the rumen fluid was promptly measured using a pH detector (FE28-standard, Mettler Toledo Co., China), while the NH_3_-N content was determined using a modified colorimetric technique (Feng and Gao, 2010). Subsequently, the rumen fluid was promptly transferred into liquid nitrogen and stored at −80°C for the analysis of rumen microbiota.

The rumen tissue was washed with saline and subsequently immersed in 4% paraformaldehyde. Subsequently, paraffin sections were prepared by removing the samples from the paraformaldehyde and rinsing them under running water for 24 h. The paraffin sections were observed under an electron microscope (ECLIPSE E200, Nikon Instruments Inc., Japan) and then photographed using a microscope image processing system (TL-507). The length and width of the rumen papilla, along with the thickness of the rumen muscular wall, were measured using ImageJ software.

#### 2.4.3 Blood parameters

Blood routine and serum biochemical indices were collected from the sheep on the 1st and 90th days of the experimental period, prior to morning feeding using conventional blood collection methods. For the blood routine, samples were drawn from blood tubes containing EDTA with 5 mL of blood. The samples were mixed and the total leukocyte count, hemoglobin concentration, and platelet count were determined using a fully automated blood cell analyzer for animals (BC-2800Vet, Mindray Co., China). Serum biochemical indices were collected from standard blood tubes containing 5 mL of blood. The tubes were allowed to stand for 40 min before being centrifuged for 15 min. Following centrifugation, the supernatant was transferred to 1.5 mL tubes for the determination of urea nitrogen and creatinine concentration using an automatic biochemical analyzer (Hitachi 7020 automatic analyzer, Hitachi Ltd., Japan).

### 2.5 High-throughput gene sequencing

The rumen samples underwent total genomic DNA extraction using the TGuide S96 Magnetic Soil/Stool DNA Kit (TIANGEN Biotech, Beijing Co., Ltd.), following the manufacturer’s protocol. The quality and quantity of the extracted DNA were assessed via electrophoresis on a 1.8% agarose gel and quantified using a NanoDrop 2000 UV–Vis spectrophotometer (Thermo Scientific, Wilmington, USA). For amplification of the full-length 16S rRNA gene, primer pairs 27F: AGRGTTTGATYNTGGCTCAG and 1492R: TASGGHTACCTTGTTASGACTT were utilized. The Internal Transcribed Spacer Identification (ITS) region was amplified using primer pairs ITS1-F: CTTGGTCATTTAGAGGAAGTAA and ITS4: TCCTCCGCTTATTGATATGC. Following quantification, amplicons with equimolar concentrations were pooled and sequenced on the PacBio Sequel II platform (Beijing Biomarker Technologies Co., Ltd., Beijing, China).

### 2.6 Bioinformatic analysis

#### 2.6.1 Operational taxonomic unit (OTU) generation process

The raw reads obtained from sequencing underwent filtering and demultiplexing using SMRT Link software (v8.0), with parameters set to minPasses ≥5 and minPredictedAccuracy ≥0.9, to obtain circular consensus sequencing (CCS) reads. Subsequently, Lima software (v1.7.0)^1^ was employed to assign the CCS sequences to the corresponding samples based on their barcodes. CCS reads devoid of primers and those falling outside the length range of 1,200–1,650 bp were discarded, utilizing forward and reverse primers identification and quality filtering via Cutadapt (v2.7, Martin, 2011). The UCHIME algorithm (v8.1)^2^ was utilized to detect and eliminate chimera sequences, resulting in obtaining clean reads. Sequences with a similarity of >97% were clustered into the same operational taxonomic unit (OTU) using USEARCH (v10.0, Edgar, 2013), and OTUs with counts less than 2 in all samples were filtered out.

#### 2.6.2 Statistical analysis

The data regarding growth performance, rumen fermentation, and blood parameters, represented as mean values, were gathered and analyzed using one-way ANOVA in SPSS (v26.0). Multiple comparisons were carried out utilizing the LSD method subsequent to identifying significant differences, with a significance level set at *p* < 0.05.

Taxonomy annotation of the OTUs was performed based on the Naive Bayes classifier in QIIME2 (Bolyen et al., 2019) using the SILVA database (Quast et al., 2013) with a confidence threshold of 70%. Alpha diversity was assessed to determine the species diversity complexity of each sample using QIIME2 software. Beta diversity calculations were conducted via Non-Metric Multi-Dimensional Scaling (NMDS) to evaluate the diversity in samples for species complexity. To investigate the influence on microbial communities, metastats analysis (White et al., 2009) was performed with R (v3.1.1). The R package of psych (v2.1.9), igraph (v1.2.5) and visNetwork (v2.1.0) we used for drafting the correlation network diagram.

## 3 Results

### 3.1 Effect of P.SMS on the growth performance of Hu sheep

ADG, DMI, and F/G of five groups during the experimental period are presented in Table 3. The results indicated that there were no significant differences in ADG and DMI among the Hu sheep across the five groups. The F/G ratios for the groups were 7.94, 8.08, 8.00, 8.89, and 9.16, respectively; notably, the F/G ratio in the PSMS20 group was a significantly higher value than that in the control group (*p* < 0.05; Table 3).

**Table 3:** Effects of dietary P.SMS supplementation on the growth performance of Hu sheep throughout the experimental period.

Items	Groups^*^					*p*-value
	CON	PSMS5	PSMS10	PSMS15	PSMS20	
Initial weight/kg	29.97	29.50	30.52	29.78	29.64	0.514
Final weight/kg	48.23	47.73	47.81	46.08	46.79	0.640
ADG/(g·d^−1^)	202.90	202.51	207.81	181.61	196.21	0.475
DMI/(g·d^−1^)	1593.70	1619.65	1612.36	1609.83	1625.85	0.457
F/G	7.94^b^	8.08^ab^	8.00^ab^	8.89^ab^	9.16^a^	0.045

* P.SMS replaced WPCS at levels of 0%, CON; 5%, PSMS5; 10%, PSMS10; 15%, PSMS15; or 20%, PSMS20.^a,b^Different letters indicate significant differences between different groups (*p* < 0.05).

### 3.2 Effect of P.SMS on rumen fermentation of Hu sheep

**Figure 1:**
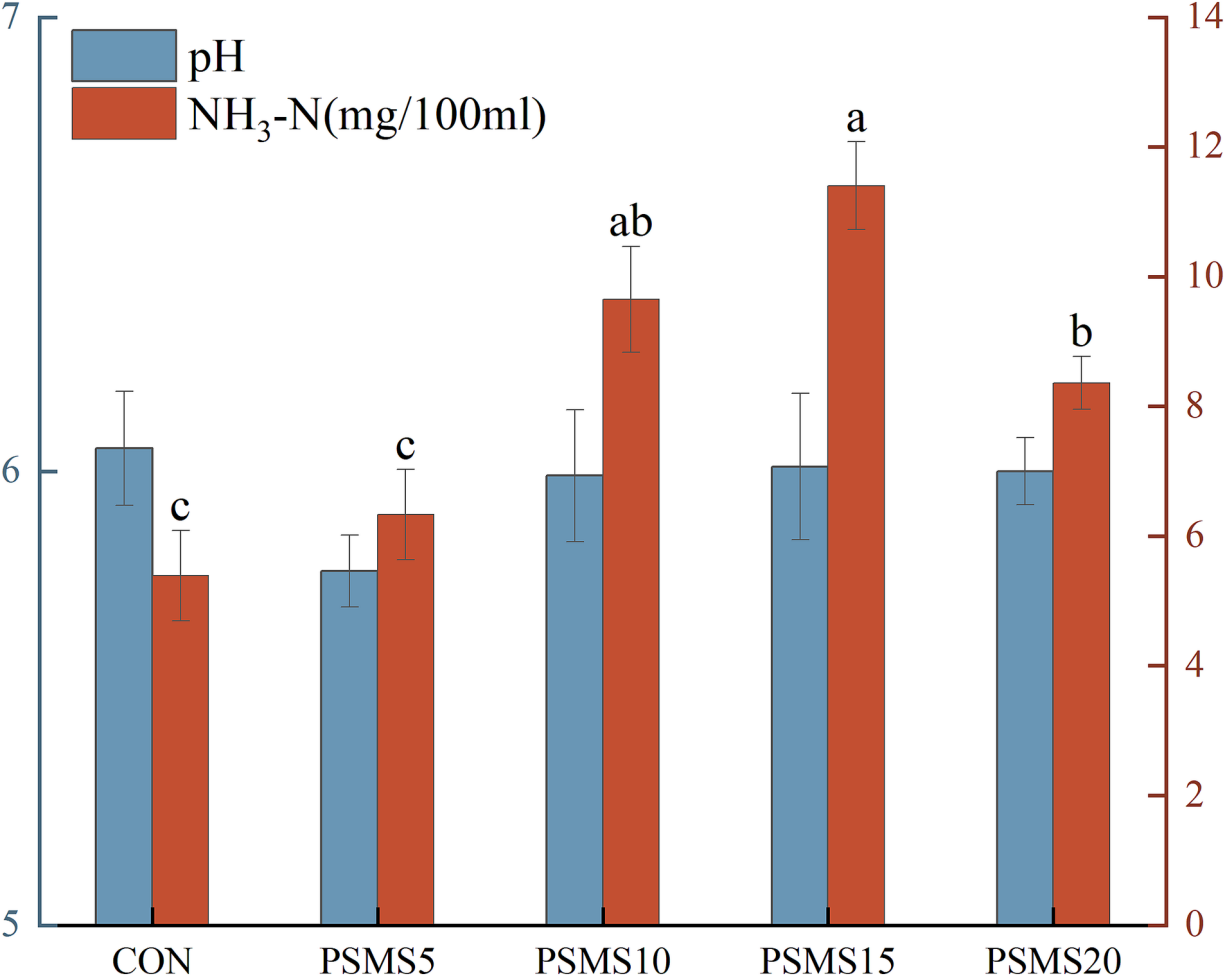
Effects of different proportions of P.SMS on rumen pH and NH_3_-N in sheep. The labels ‘a,’ ‘b,’ and ‘c’ above the bars indicate significant differences between different groups (*p <* 0.05). The absence of labels indicates no significant difference. P.SMS replaced WPCS at levels of 0%, CON; 5%, PSMS5; 10%, PSMS10; 15%, PSMS15; or 20%, PSMS20.

As illustrated in Figure 1, the NH_3_-N concentration in PSMS15 was significantly higher than that in CON, PSMS5, and PSMS20 (*p* < 0.05). Additionally, the NH_3_-N concentration in PSMS10 and PSMS20 was significantly elevated compared to CON and PSMS5 (*p* < 0.05).

### 3.3 Effect of P.SMS on rumen histomorphology of Hu sheep

Table 4 presents the measurements of rumen papilla length and width, as well as rumen muscular thickness. The results indicate an increase in both the length and width of the rumen papilla with increasing proportions of P.SMS. Specifically, PSMS15 exhibited a significantly greater rumen papilla length compared to both CON and PSMS5 (*p* < 0.05). Moreover, PSMS15 and PSMS20 demonstrated significantly wider rumen papillae compared to CON and PSMS5 (*p* < 0.05).

**Table 4:** Effects of P.SMS on rumen tissue morphology in Hu sheep.

Items	Groups^*^					*p*-value
	CON	PSMS5	PSMS10	PSMS15	PSMS20	
Rumen papilla length/mm	2.49^b^	2.48^b^	2.59^ab^	2.61^ab^	2.73^a^	0.027
Rumen papilla width/mm	0.42^b^	0.42^b^	0.45^ab^	0.46^a^	0.489^a^	0.017
Rumen muscular thickness	3.83	3.90	4.02	3.98	4.00	0.135

* P.SMS replaced WPCS at levels of 0%, CON; 5%, PSMS5; 10%, PSMS10; 15%, PSMS15; or 20%, PSMS20.^a,b^Different letters indicate significant differences between different groups (*p* < 0.05).

### 3.4 Effect of P.SMS on blood parameters of Hu sheep

**Figure 2:**
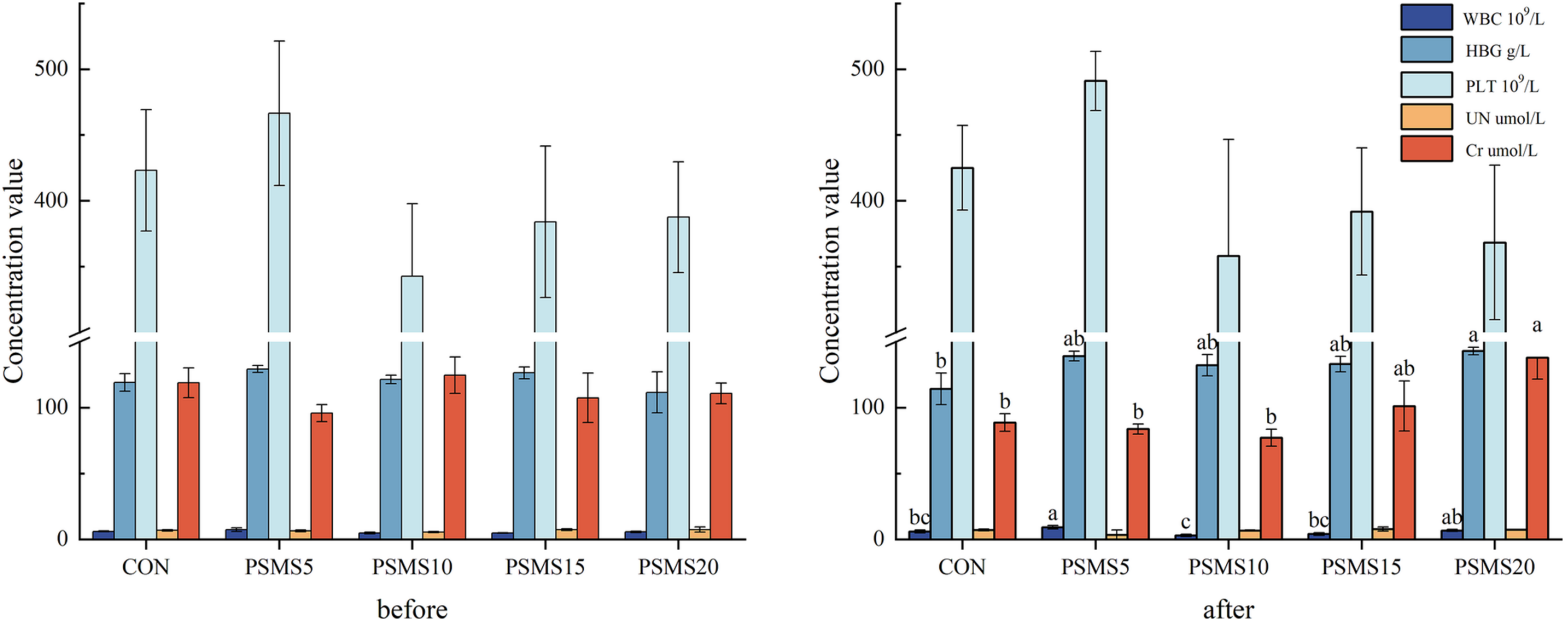
Selected blood parameters before and after feeding P.SMS. The labels ‘a’, ‘b’, and ‘c’ above the bars indicate significant differences between different groups (*p <* 0.05). The absence of labels indicates no significant difference. WBC, white blood cell; HGB, hemoglobin concentration; PLT, Platelet count; UN, urea nitrogen; Cr, creatinine. P.SMS replaced WPCS at levels of 0%, CON; 5%, PSMS5; 10%, PSMS10; 15%, PSMS15; or 20%, PSMS20.

Figure 2 presents the hematological and serological parameters before and after feeding throughout the experiment, highlighting significant differences in white blood cell count, hemoglobin concentration, and creatinine levels following P.SMS feeding. Notably, white blood cell counts were higher in PSMS5 and PSMS20 compared to PSMS10, with a significant increase in PSMS5 relative to both CON and PSMS15 (*p* < 0.05). Additionally, hemoglobin concentration exhibited a significant increase in PSMS20 compared to CON (*p* < 0.05). Furthermore, creatinine levels were significantly elevated in PSMS20 compared to CON, PSMS5, and PSMS10 (*p* < 0.05).

### 3.5 Sequencing data quality assessment

The raw data were processed according to the aforementioned method, and data quality was assessed by evaluating factors such as read length and read counts at each stage, as detailed in Supplementary Table 1. The sequencing results for bacterial and fungal samples revealed the following ranges for clean circular consensus sequences (CCS), sequence effectiveness, and sequencing lengths: 6713–8,451 and 4,078–8,417; 94.84–98.98% and 99.13–99.95%; 1,454–1,459 bp and 593–653 bp, respectively.

### 3.6 Impact of P.SMS addition on ruminal microbial community in Hu sheep

#### 3.6.1 Impact on microbial community structure

**Figure 3:**
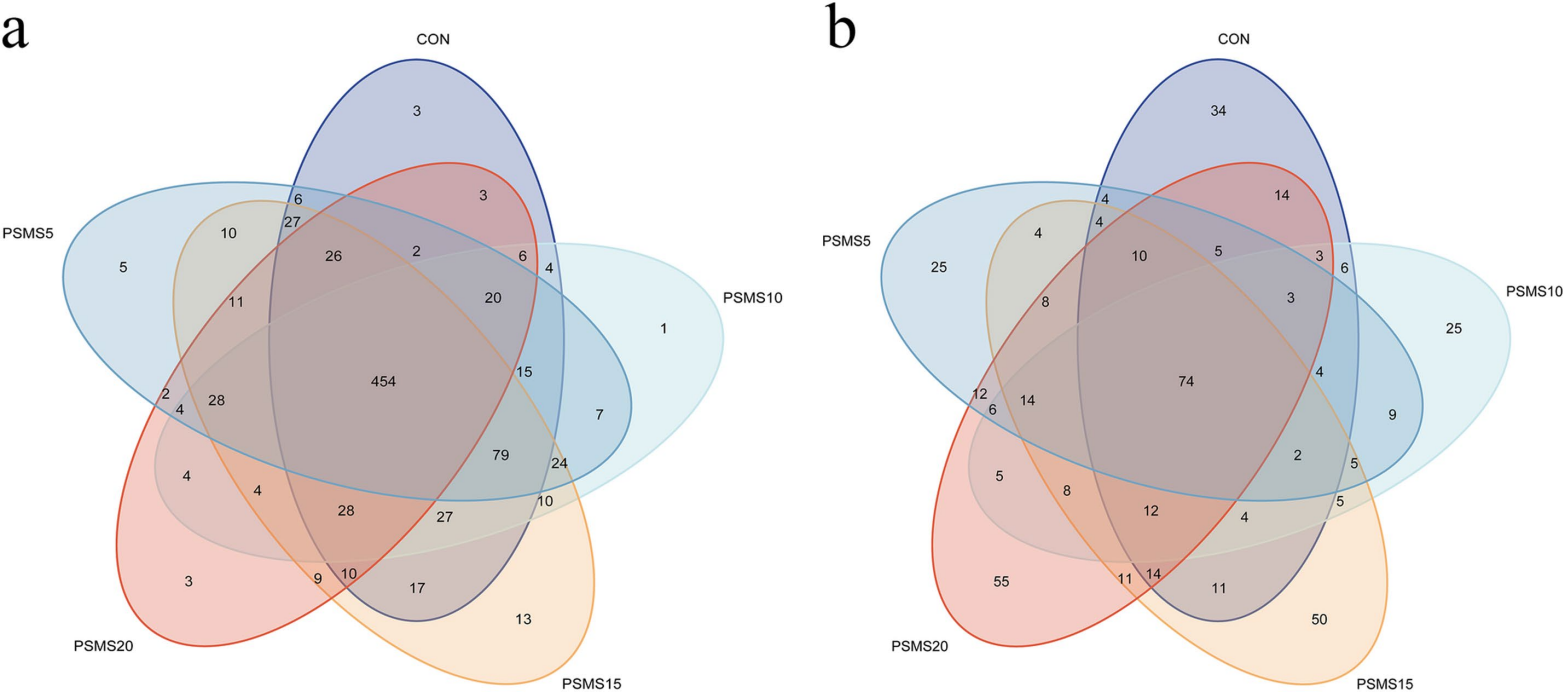
Venn diagram of OTUs in (a) rumen bacteria and (b) fungi of Hu sheep. The different colors of the ellipses in the Venn diagram represent different groups, and the overlapping regions indicate the number of shared features among the groups. The non-overlapping regions represent the number of features specific to each group. P.SMS replaced WPCS at levels of 0%, CON; 5%, PSMS5; 10%, PSMS10; 15%, PSMS15; or 20%, PSMS20.

Based on the analysis of 97% of nucleotide sequences from a total of 90,133 bacterial reads, 862 Operational Taxonomic Units (OTUs) were identified (Figure 3a). Among these, 454 OTUs were common to all five groups, representing 52.67% of the total OTUs. The number of unique OTUs in CON, PSMS5, PSMS10, PSMS15, and PSMS20 groups were 3 (0.35%), 5 (0.58%), 1 (0.12%), 13 (1.51%), and 3 (0.35%), respectively. PSMS15 exhibited the highest number of OTUs, whereas PSMS20 showed the lowest.

For fungal sequencing, a total of 178,688 reads identified 446 OTUs (Figure 3b). Among these, 74 OTUs were shared among all five groups, accounting for 16.59% of the total OTUs. The unique OTU counts in CON, PSMS5, PSMS10, PSMS15, and PSMS20 were 34 (7.62%), 25 (5.61%), 25 (5.61%), 50 (11.21%), and 55 (12.33%), respectively. PSMS20 displayed the highest number of OTUs, while CON had the lowest.

#### 3.6.2 Alpha diversity analysis

Table 5 indicates that the bacterial and fungal indices did not exhibit significant differences among the groups. The Fungal ACE index and Chao1 index demonstrated a pattern of initially decreasing and then increasing (*p* > 0.05), with the fungal group in PSMS10 displaying the lowest abundance of the fungal community. A closer inspection of the table, reveals that fungal indices showed a decreasing trend in both the abundance and diversity of the fungal community from CON to PSMS5. However, as community abundance decreased, community diversity increased from PSMS5 to PSMS10. The increase in community abundance from PSMS5 to PSMS10 and PSMS15 was accompanied by a trend toward flattening community diversity. Conversely, the trend of bacterial diversity in the rumen was opposite to that of fungi. Notably, the decrease in colony abundance from CON to PSMS5 was associated with an increase in colony diversity. The trends in bacterial colony abundance and colony diversity were consistent across the other groups.

**Table 5:** Effects of P.SMS on Alpha diversity of rumen bacteria and fungi in Hu sheep.

Items	Groups^*^					*p-*value
	CON	PSMS5	PSMS10	PSMS15	PSMS20	
Bacteria						
ACE	573	536.15	530.34	569.34	446.66	0.291
Chao1	517.81	506.02	493.68	552.79	406.4	0.312
Simpson	0.90	0.97	0.94	0.97	0.92	0.132
Shannon	5.75	6.47	6.00	6.72	5.53	0.270
PD_whole_tree	30.44	28.82	28.00	29.19	22.44	0.352
Coverage	0.96	0.96	0.96	0.96	0.96	
Fungus						
ACE	126.60	97.55	89.22	97.27	105.06	0.297
Chao1	126.73	94.05	85.38	98.78	108.07	0.455
Simpson	0.90	0.87	0.91	0.87	0.87	0.319
Shannon	4.19	3.96	4.18	4.00	3.99	0.643
PD_whole_tree	18.19	19.31	18.75	20.39	21.15	0.596
Coverage	1.00	1.00	1.00	1.00	1.00	

* P.SMS replaced WPCS at levels of 0%, CON; 5%, PSMS5; 10%, PSMS10; 15%, PSMS15; or 20%, PSMS20.

#### 3.6.3 Beta diversity analysis

**Figure 4:**
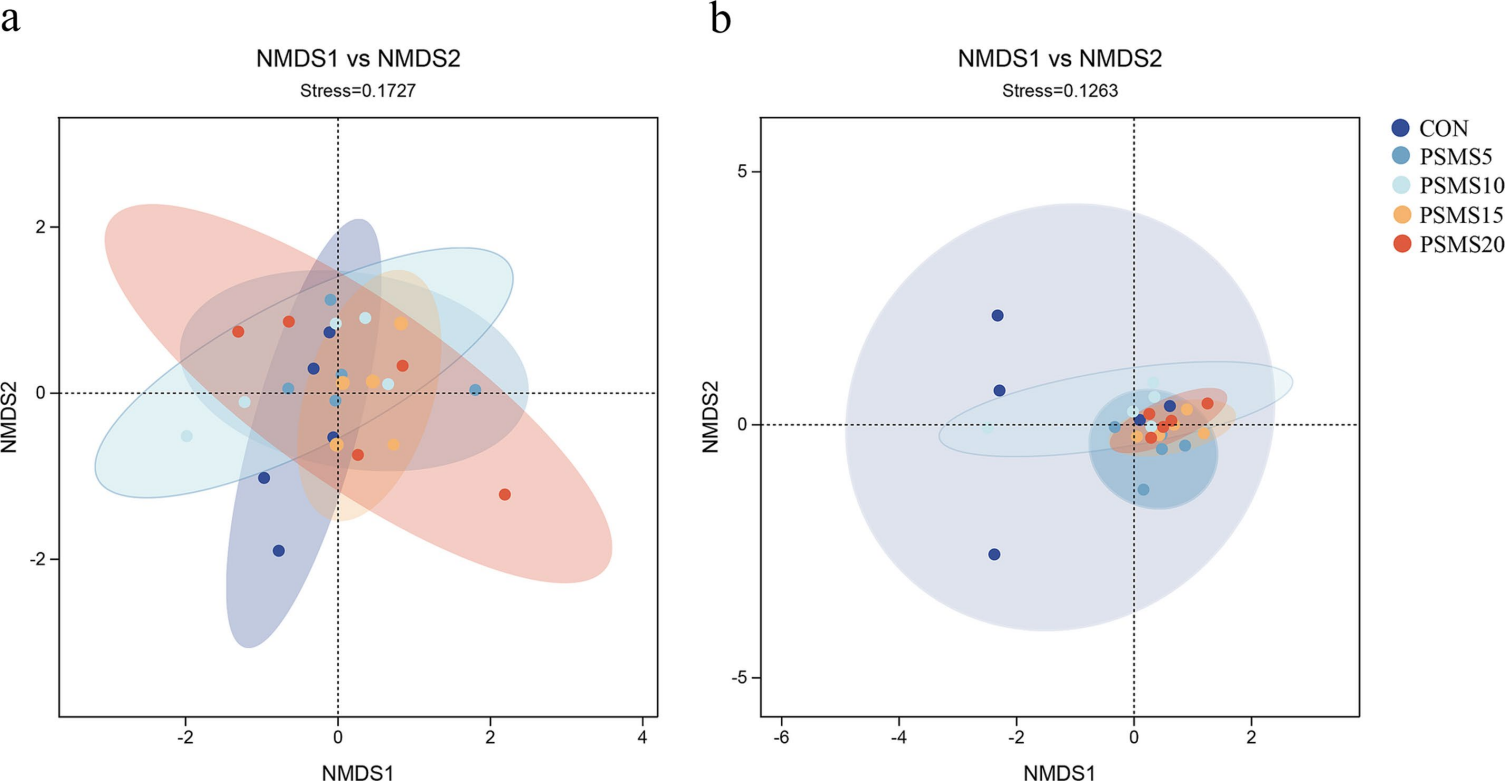
NMDS analysis of (a) rumen bacteria and (b) fungi of Hu sheep. Each point in the figure represents a sample; different colors represent different subgroups; the oval circle indicates that it was a 95% confidence ellipse. When Stress is less than 0.2, it indicates that the NMDS analysis has some reliability. The closer the samples are to each other on the coordinate graph, the higher the similarity. P.SMS replaced WPCS at levels of 0%, CON; 5%, PSMS5; 10%, PSMS10; 15%, PSMS15; or 20%, PSMS20.

As depicted in Figure 4, both stress values in the NMDS analyses are below 0.2, indicating a relatively reliable NMDS analysis. Both analyses revealed no significant differences in the distribution of bacterial and fungal microbiota among the groups. However, there appeared to be a tendency for the distribution of fungal communities to cluster, particularly between PSMS15 and PSMS20.

#### 3.6.4 Composition of groups at the level of phylum and genus classification

**Figure 5:**
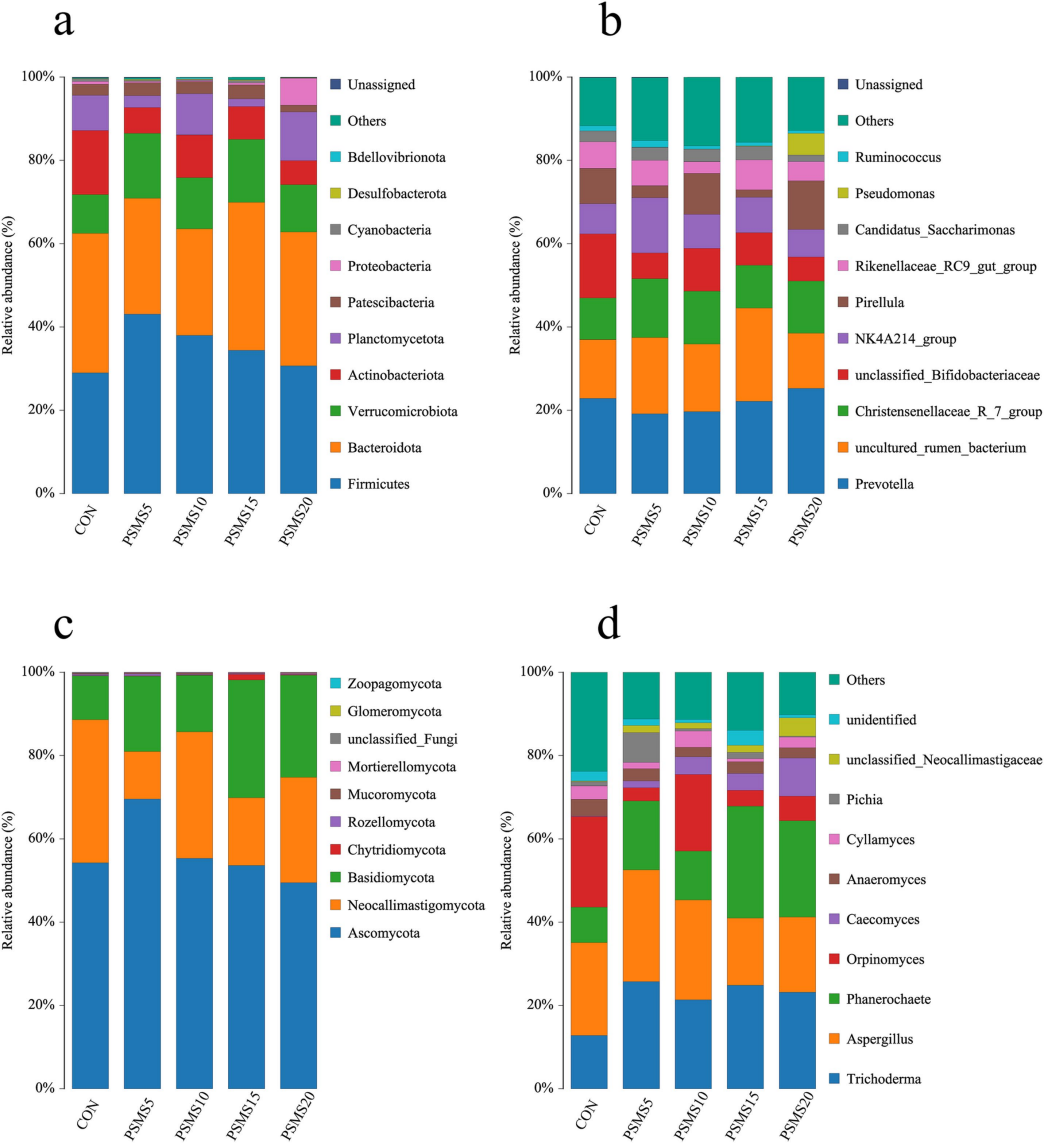
The relative abundance of ruminal bacteria at the level of (a) phylum and (b) genus. The relative abundance of ruminal fungi at the level of (c) phylum and (d) genus. The horizontal axis in the figure represents group names, and the vertical axis represents relative abundance percentages. Different colors represent different species, and stacked bars indicate the top 10 taxonomic groups of relative abundance at each classification level. P.SMS replaced WPCS at levels of 0%, CON; 5%, PSMS5; 10%, PSMS10; 15%, PSMS15; or 20%, PSMS20.

The dominant organisms at the phylum level of rumen microbiota in all five groups of Hu sheep were *Firmicutes* and *Bacteroidetes*, with relative abundances ranging from 63.10 to 72.28% (Supplementary Table 3). Notably, as illustrated in Figure 5a and Supplementary Table 3 *Firmicutes* displayed an increasing trend followed by a decrease across the groups. PSMS5 had the highest abundance, while PSMS20 exhibited the lowest (*p* > 0.05). In contrast, *Bacteroidetes* showed a decreasing trend followed by an increase across the groups, with PSMS20 demonstrating the highest abundance and PSMS10 the lowest (*p* > 0.05). The ratio of *Firmicutes* to *Bacteroidetes* across the five groups was 0.94, 1.37, 1.53, 1, and 0.81, indicating an upward trend followed by a downward trend, as shown in Supplementary Table 3. At the genus level, as depicted in Figure 5b and Supplementary Table 4, *Prevotella* and *uncultured_rumen_bacterium* were the dominant genera, with relative abundances ranging from 36.61 to 44.47%. *Prevotella* exhibited a decreasing trend followed by an increase, with the highest abundance found in PSMS10 and the lowest in CON (*p* > 0.05). The *Christensenellaceae_R_7_group* also displayed an increasing and then decreasing trend, with the highest abundance in PSMS10 and the lowest in CON (*p* > 0.05).

The predominant organisms at the phylum level of rumen fungi in all five groups of Hu sheep were *Ascomycota* and *Neocallimastigomycota*, with relative abundances ranging from 69.41 to 89.00%. Notably, Ascomycota displayed a trend of initially increasing and then decreasing with the addition of P.SMS, peaking in PSMS5 and reaching its lowest point in PSMS20 (*p* > 0.05). (Supplementary Table 3 and Figure 5c). Furthermore, Supplementary Table 3 indicates that the percentage of *Basidiomycota* was significantly higher in PSMS15 and PSMS20 compared to CON, and significantly higher in PSMS15 than in PSMS10 (*p* < 0.05). Additionally, *Mucoromycota* showed an initial increase followed by a subsequent decrease, with PSMS10 exhibiting the highest percentage and CON the lowest (*p* > 0.05). At the genus level, as illustrated in Figure 5d and Supplementary Table 4, *Trichoderma* and *Aspergillus* emerged as the dominant genera, with relative abundances ranging from 33.35 to 52.84%. According to Supplementary Table 4, the percentage of *Phanerochaete* was significantly higher in PSMS15 compared to CON and PSMS10, while both PSMS15 and PSMS20 were significantly higher than CON, PSMS5, and PSMS10 (*p* < 0.05).

#### 3.6.5 Impact on the difference in microbial community

**Figure 6:**
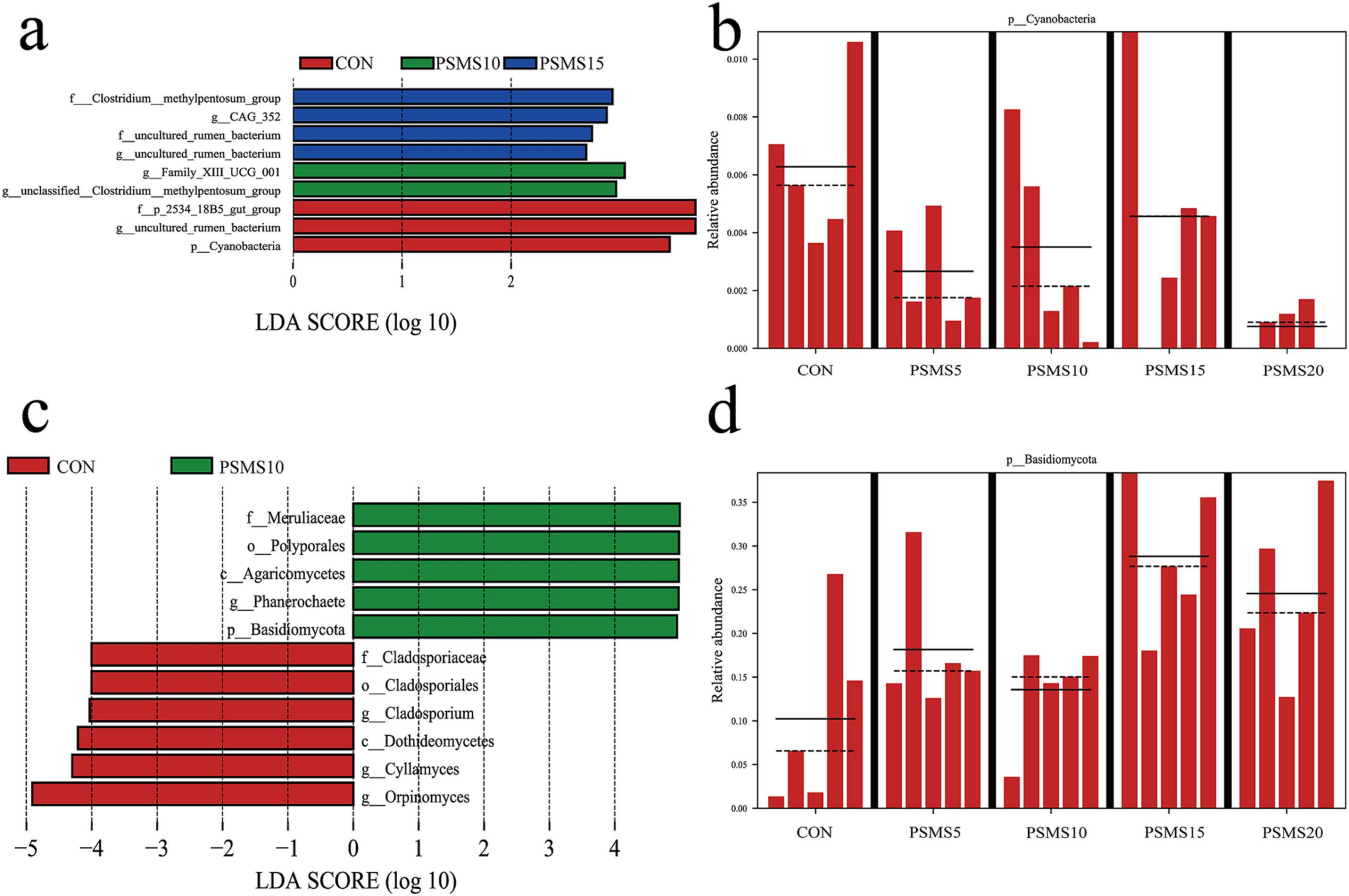
LEfSe analysis of rumen bacteria and fungi. Linear discriminant analysis effect size cladogram based on classification of (a) rumen bacteria and (c) fungi. Comparison of the relative abundance of (b) bacterial and (d) fungal biomarkers at the phylum level between five groups. P.SMS replaced WPCS at levels of 0%, CON; 5%, PSMS5; 10%, PSMS10; 15%, PSMS15; or 20%, PSMS20.

LEfSe analysis of the rumen microbiota revealed the dominance of specific bacterial and fungal groups, as illustrated in Figure 6. Figures 6a,c highlighted the presence of 7 bacterial and 11 fungal differences observed at various taxonomic levels between the CON and PSMS15 groups. At the phylum level, *Cyanobacteria* and *Basidiomycota* were identified as biomarkers distinguishing CON from PSMS15. Figures 6b,d provide a comparison of the distribution of these two phyla across different groups. The *Cyanobacteria* phylum was found to be relatively abundant in the CON group, whereas the *Basidiomycota* phylum was more prevalent in PSMS15.

### 3.7 Bacterial-fungal interactions in Hu sheep rumen

**Figure 7:**
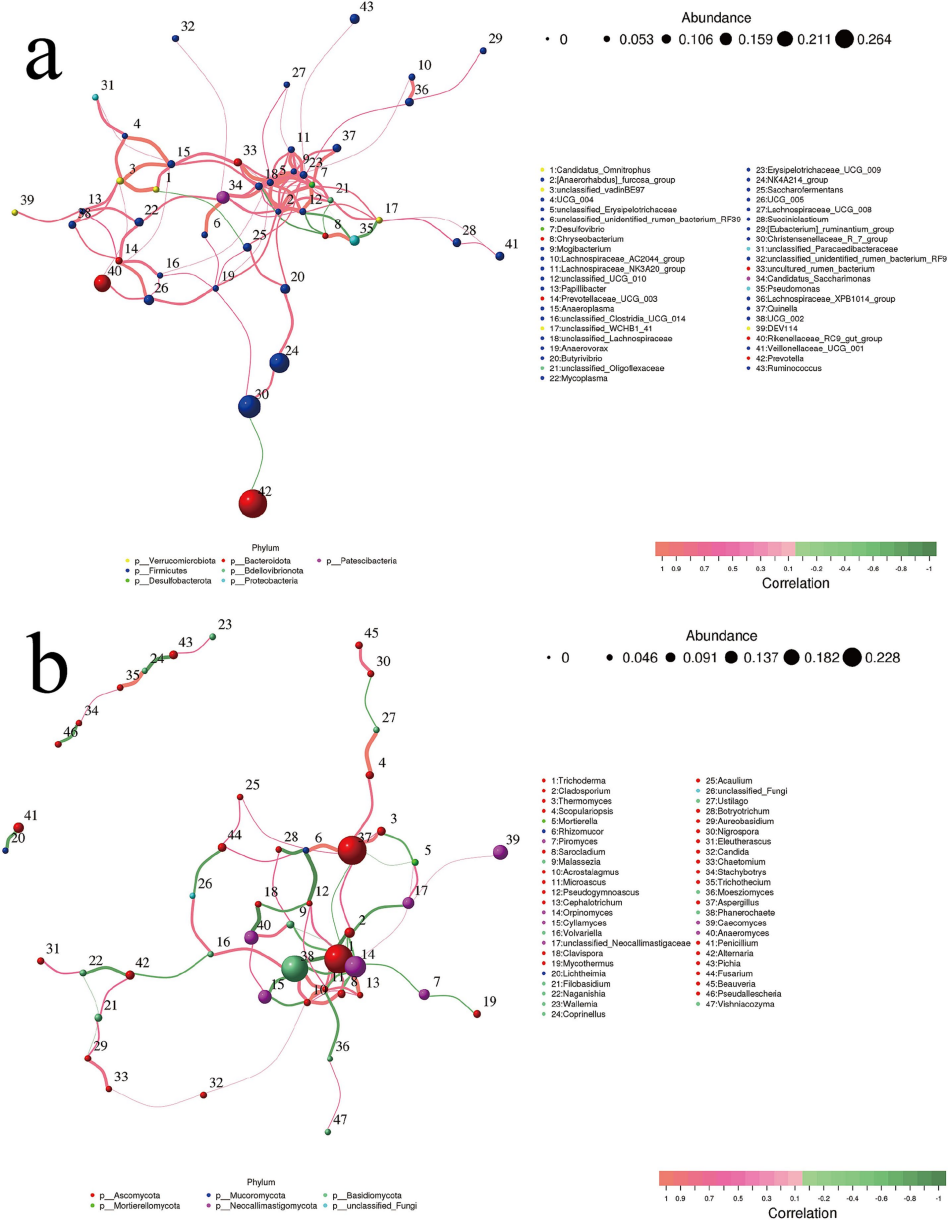
Correlation network of top 50 genera for (a) rumen bacteria and (b) fungi. Species are represented by circles, where the size of each circle indicates the abundance of the species. Furthermore, different colors are used to denote distinct phylum classifications. Edges represent the correlation between two species, with line thickness indicating strength and line color indicating correlation direction: orange for positive correlation and green for negative correlation. P.SMS replaced WPCS at levels of 0%, CON; 5%, PSMS5; 10%, PSMS10; 15%, PSMS15; or 20%, PSMS20.

In Figure 7a, the majority of bacterial genera at the center of the association are classified within the *Firmicutes* phylum, while the *Desulfovibrio* genus (NO. 7) from the *Desulfobacterota* phylum also occupies a central position. The figure primarily illustrates a positive correlation. After excluding undefined and uncultured bacteria, we observed the presence of two pairs of weaker negative correlations: one pair consists of *Candidatus Omnitrophus* (NO. 1) and *Saccharofermentans* (NO. 25), while the other pair includes *Prevotella* (NO. 42) and *Christensenellaceae_R_7_group* (NO. 30).

In Figure 7b and Supplementary Figure 1, two distinct layout arrangements of the correlation analysis network are presented. The main interactions are concentrated among three phyla: *Ascomycota*, *Basidiomycota*, and *Neoxallimastigomycota*. Specifically, *Trichoderma* (NO. 1) and *Aspergillus* (NO. 37) are members of *Ascomycota*, *Phanerochaete* (NO. 38) belongs to *Basidiomycota*, and *Orpincomycota* (NO. 14) is part of *Neoxallimastigomycota*, all of which contribute to the core interactions. Two clusters of fungi show independent correlations: one between *Ascomycota* and *Basidiomycota*, and the other between *Ascomycota* and *Mucoromycota* (Figure 7b).

**Figure 8:**
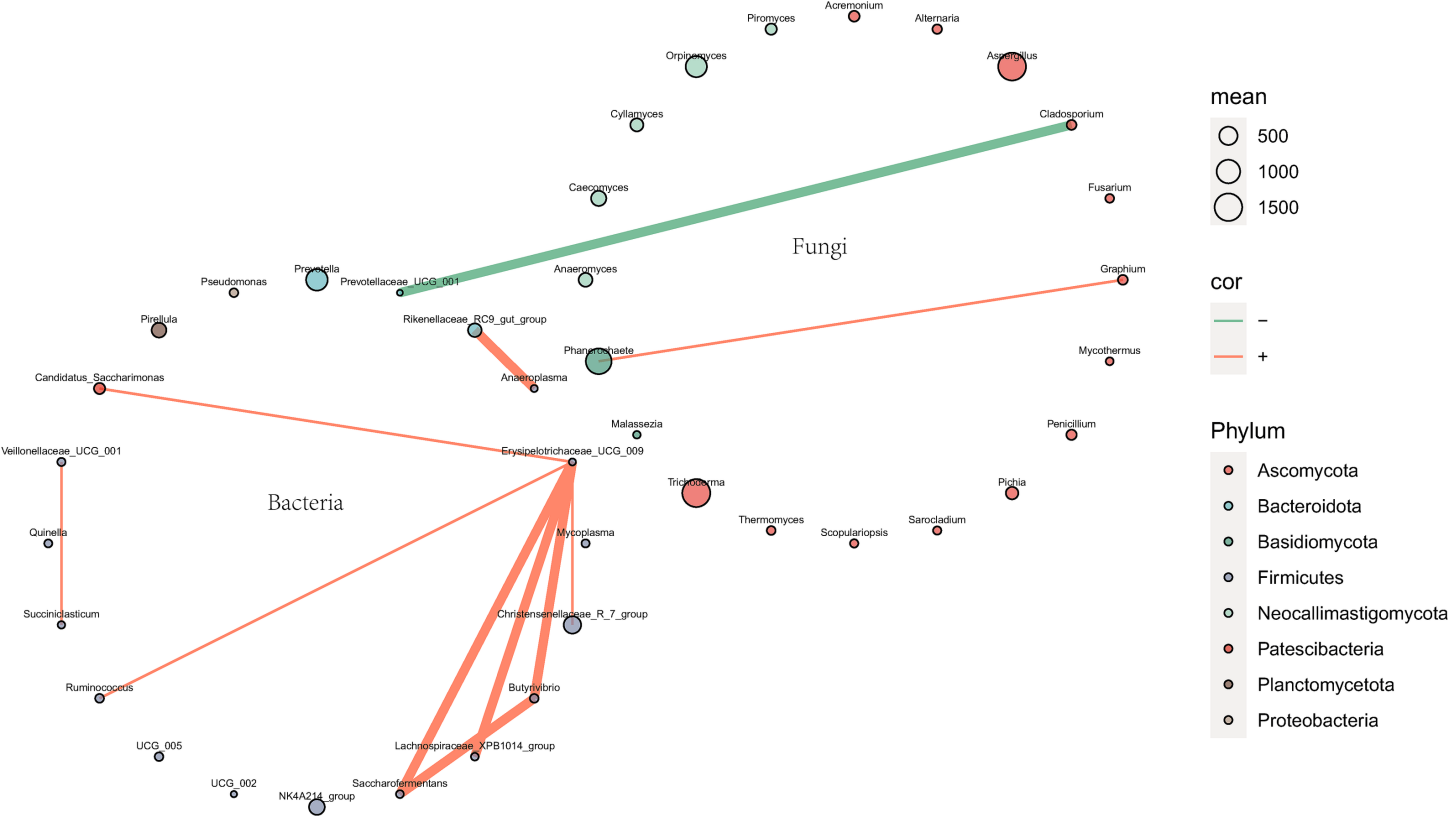
Correlation network of top 20 genera between rumen bacteria and fungi. Species are represented by circles, where the size of each circle indicates the abundance of the species. Furthermore, edges represent the correlation between two species, with a width of 2 when the absolute value of correlation >0.65 and a width of 0.5 otherwise. The line color indicates correlation direction: orange for positive correlation and green for negative correlation. P.SMS replaced WPCS at levels of 0%, CON; 5%, PSMS5; 10%, PSMS10; 15%, PSMS15; or 20%, PSMS20.

The correlation network of the top 20 genera between rumen bacteria and fungi is illustrated in Figure 8, which reveals a statistically significant negative correlation between *Cladosporium* and *Prevotellaceae_UCG_001,* with an absolute correlation coefficient exceeding 0.65.

## 4 Discussion

Each kilogram of mushroom production yields approximately five kilograms of SMS (Lucas De Jesus et al., 2023). SMS is classified as agricultural waste and is often either landfilled, incinerated, or disposed of improperly, which prevents the full utilization of its untapped nutrients and mycelium (Huang et al., 2023; Yang et al., 2022). However, the implementation of reuse or recycling strategies could significantly reduce this solid waste, generating new economic opportunities and promoting positive environmental outcomes (Huang et al., 2023). In addition to the residual nutrients in SMS, mushroom polysaccharides present in the fermentation liquid of edible fungi, mycelium, or fruit bodies contribute to regulating the gut microbiome, enhancing the intestinal mucosal barrier, modulating lipid metabolism, and increasing the production of short-chain fatty acids (Zhao J. et al., 2023). While some studies have explored the utilization of SMS, limited attention has been paid to its effects on rumen bacterial composition and the relationships between rumen bacteria and fungi. In our study, varied results were observed as the percentage of substitutions processed SMS (P.SMS) changed.

In this experiment, the F/G was significantly higher in the PSMS20 group compared to the CON group, while ADG and DMI did not differ among the five groups. This finding indicates a lower feed utilization efficiency when a high percentage of P.SMS was fed. Hanlon et al. (2023) noted that nutrient synchronization of protein and carbohydrates is an effective strategy for enhancing rumen nutrient utilization. The F/G ratio of PSMS20 was significantly greater than that of the CON group. This may be attributed to the fact that, although the protein content increased with the proportion of P.SMS, the efficiency of nutrient utilization decreased due to the lower soluble carbohydrate content of P.SMS compared to whole-plant corn silage. PSMS15 exhibited a significantly higher NH_3_-N concentration compared to CON, PSMS5, and PSMS20, while PSMS10 and PSMS20 also demonstrated significantly higher NH_3_-N concentrations than CON and PSMS5. The NH_3_-N content is influenced by the rate of microbial degradation of nitrogenous substances in the ration, as well as the rate of NH_3_-N utilization by microorganisms and rumen epithelial cells (Liang et al., 2024). Rehemujiang et al. (2023) indicated that the normal physiological range of rumen NH_3_-N is 6-30 mg/dL; all groups were within this normal range except for the CON group, which had NH_3_-N levels exceeding the minimum concentration required for the development of rumen microorganisms, which is 5 mg/dL. Despite the consistent nitrogen content in diets formulated according to isoenergetics and isonitrogen principles, variations were observed in the microbial and rumen epithelial utilization rates, as well as in the composition of bacteria and fungi at both the phylum and genus levels. These variations may contribute to differences in the NH_3_-N concentrations. Shen et al. (2023) noted a positive correlation between rumen fluid pH and its free ammonia nitrogen (FAN), along with a strong negative correlation between FAN and microbial population. However, there were no significant differences in rumen fluid pH among the five groups, suggesting that there may also be no significant difference in FAN among these groups. Rumen papillae length and width, along with rumen musculature thickness, are key indicators of rumen development (Lesmeister et al., 2004). In this experiment, with the exception of PSMS5, both the length and width of the rumen papillae increased with higher proportions of P.SMS added, indicating that the inclusion of P.SMS can promote rumen development in sheep. Gadelha et al. (2014) reported that monogastric animals, including birds, fish, pigs, and rodents, exhibit greater susceptibility to gossypol toxicity compared to ruminants; these effects tend to accumulate over a period of 1 to 3 months of ingestion. This was further corroborated by blood parameters observed in this experiment, where Cr content was significantly higher in PSMS20 compared to CON, PSMS5, and PSMS10, and the concentration of HGB was significantly higher in PSMS20 than in CON. Serum Cr levels reflect the animal’s utilization of proteins and amino acids, and a high serum Cr level may indicate impairment of kidney function (Wong Vega et al., 2020). The presence of gossypol may also affect the utilization of Fe^2+^ ions in the blood, potentially impacting the function of HGB as an oxygen transport carrier, leading to alterations in oxygen-carrying capacity (Gadelha et al., 2014).

In the analysis of bacterial composition in this experiment, changes were observed in the phyla *Bacteroidetes* and *Firmicutes*, indicating shifts in substrate utilization patterns. This microbiome investigation revealed that *Bacteroidetes* and *Firmicutes* were the dominant bacterial phyla, consistent with previous studies (Chen et al., 2022; Cui Y. et al., 2022; Han et al., 2019; Pinnell et al., 2022). These two phyla serve as indicators for assessing the energy needs of ruminants (Xue et al., 2017), with *Bacteroidetes* being responsible for acetate and propionate production, while *Firmicutes* are responsible for butyrate production (Cui Y. et al., 2022). The *Firmicutes/Bacteroidetes* ratio across the five groups demonstrated a clear trend of change, indicating significant structural alterations related to substrate utilization. Gharechahi and Salekdeh (2018) found that *Bacteroidetes*-associated species exhibited a high abundance of genes encoding debranching and oligosaccharide-degrading enzymes, whereas *Firmicutes*-associated species showed a richness in cellulases and hemicellulases. White et al. (2014) pointed that *Firmicutes* utilize cell surface-associated cellulose-degrading complexes, while *Bacteroidetes* employ outer membrane binding proteins encoded by gene clusters to degrade soluble polysaccharides primarily within the periplasmic or intracellular space. The transition from *Bacteroidetes* being the dominant phylum to *Firmicutes*, as observed in the experiment conducted by Xue et al. (2022), was attributed to a decreased level of dietary physically effective neutral detergent fiber (peNDF). P.SMS was provided in a crushed state while WPCS was fed in its original form, the crushed P.SMS exhibited a lower NDF compared to the original WPCS (Tables 1, 2). The experiment conducted by Palangi et al. (2022) also demonstrated a significant reduction in the acid detergent fiber and crude fiber contents of the substrate following mushroom production. This difference may contribute to the observed transition of the dominant phylum from *Bacteroidetes* to *Firmicutes* as the proportion of P.SMS from 0 to 10%, aligns with the findings of Xue et al. (2022). Ma G. et al. (2022) reported that the polysaccharide from *Pleurotus eryngii* significantly promoted the relative abundance of *Firmicutes* while inhibiting that of *Bacteroidetes*, suggesting a similar effect may occur with the polysaccharide in *Pleurotus ostreatus.* Furthermore, as the proportion of P.SMS continued to increase, the dominant phylum transitioned from *Firmicutes* to *Bacteroidetes* in PSMS15 and PSMS20. Although the level of NDF decreased with the increasing proportion of P.SMS in the diet, there was a gradual increase in the content of gossypol in cottonseed hulls, which may contribute to this transition. Wang et al. (2021) noted that low concentrations of gossypol enhanced the abundance of rumen bacteria involved in fiber and starch metabolism; however, gossypol at high concentrations (>0.5 mg/g) exhibited an inhibitory effect on the activity of rumen microbes, including six genera within the *Firmicutes* phylum. This conclusion, consistent with the current experimental findings, reflects both the significantly higher NH_3_-N content in PSMS15 compared to CON, PSMS5, and PSMS20, and the observed transition of the major phyla from *Firmicutes* to *Bacteroidetes*. Given that the two phyla share common characteristics for the degradation of phytopolysaccharides, they may be particularly efficient in utilizing nitrogenous material at a close ratio. Xue et al. (2022) discovered in their experiment that *Prevotella* and *Christensenellaceae_R_7_group* were the dominant genera, exhibiting a positive quadratic correlation with the level of peNDF. Zhang X. et al. (2022) confirmed that *Prevotella* was indeed the dominant genus across all samples, while the relative abundance of *Christensenellaceae_R_7_group* varied significantly among the treatment groups. Other studies have also identified the prevailing status of these genera within the microbiota (Cui et al., 2021; Zhao W. et al., 2023). Numerous reports have highlighted the strong capacity of *Prevotella* for the degradation of hemicellulose (Cui Y. et al., 2022; Li et al., 2015), as it plays a crucial role in the decomposition of starch, xylan, crude proteins, and pectin (Han et al., 2019; Huang et al., 2023; Qian et al., 2019; Xue et al., 2022). *Christensenellaceae* is a prevalent bacterial family in both human and animal guts, potentially capable of metabolizing non-fiber carbohydrates (Zhang et al., 2023). Previous studies have suggested possible associations between *Christensenellaceae*, lipid metabolism, and ether extract digestibility (Shang et al., 2021; Waters and Ley, 2019; Zhang et al., 2023). The observed negative correlation between the abundance changes of *Christensenellaceae_R_7_group* and *Prevotella* may be attributed to their competition for similar nutrient resources within the gut environment. While both *Christensenellaceae_R_7_group* and *Prevotella* play significant roles in feed digestion, they exhibit different preferences for specific types of carbohydrates. Consequently, fluctuations in the abundance of one genus may influence the growth and survival of the other, leading to a negative correlation between their respective abundances.

In addition to the two phyla that account for a relatively large proportion, *Patescibacteria* and *Cyanobacteria* were also affected by the addition of P.SMS. *Patescibacteria*, also known as the candidate phyla radiation (CPR), constitute a diverse and disproportionately large fraction of microbial dark matter, which remains largely unidentified, unclassified, and understudied (Wang et al., 2023). Members of the CPR exhibit a variety of unique traits, including their small width, compact genomes typically less than 1 Mb, and limited metabolic capabilities (Ji et al., 2022; Wang et al., 2023). This has led to the hypothesis of their universal dependence on host organisms for growth, which may explain the positive correlation observed between *Erysipelotrichaceae_UCG_009* and *Candidatus_Saccharimonas*. Experimental findings support this notion, indicating that the majority of cultivated *Patescibacteria* adhere to and thrive on the surfaces of other bacteria, functioning as obligate epibionts (Batinovic et al., 2021; Kuroda et al., 2022). *Erysipelotrichaceae* are likely related to host metabolism or cholesterol phenotypes in humans and mice, while in ruminants, they may aid in the degradation of plant fibers in the rumen (Kaakoush, 2015; Qian et al., 2019; Zhang et al., 2023). The genera *Candidatus_Saccharimonas* and *Christensenellaceae_R_7_group*, as previously mentioned, demonstrated a positive correlation with metabolites involved in energy substrate metabolism and amino acid biosynthesis (Ogunade et al., 2019). Zhen et al. (2023) observed that the addition of both sodium acetate and sodium propionate resulted in increased abundances of specific rumen bacteria, including *Candidatus Saccharimonas* and *Christensenellaceae R-7*. The molar proportion of acetate significantly increased with gossypol supplementation (Wang et al., 2020), subsequently elevating the proportion of *Candidatus Saccharimonas* in PSMS5, PSMS10, and PSMS15. However, as the proportion of gossypol increased, the abundance of *Candidatus_Saccharimonas* decreased, potentially due to a negative effect of gossypol on its growth or the promotion of other bacteria that inhibited the growth of *Candidatus_Saccharimonas* in PSMS20. Furthermore, the positive correlation between *Candidatus_Saccharimonas* and the *Christensenellaceae_R_7_group* suggests a potential interaction within the gut microbiota, indicating their joint involvement in energy metabolism and nutrient synthesis. *Cyanobacteria,* commonly referred to as blue-green algae, were significantly less abundant in PSMS20 compared to other groups. The *Cyanobacteria* present in the rumen are recognized as polysaccharolytic organisms, capable of breaking down complex polysaccharides (McGovern et al., 2018). As algal populations proliferate, algal blooms can lead to adverse consequences including changes in water color, elevated biotoxin levels in aquatic organisms, and harm to both humans and other organisms (Hallegraeff et al., 2021). Previous studies have demonstrated that the enzyme systems of white-rot fungi exhibit an inhibitory effect on algae and that these fungi can utilize algae as a carbon and nitrogen source for growth and utilization (Yu et al., 2023). It is evident that a large proportion of P.SMS results in a significant reduction in *Cyanobacteria* abundance; this may be attributed to either the inhibitory effect of gossypol or the utilization of algae by the enzyme systems of white-rot fungi. The precise mechanisms underlying this phenomenon warrant further investigation.

In the realm of rumen microbiota research, a noticeable disparity exists between the number of studies conducted on rumen fungi and those on rumen bacteria. Anaerobic fungi have been established as a significant component of the population of ruminal microorganisms responsible for fiber digestion (Hartinger and Zebeli, 2021; Zhang et al., 2007). Among the fungal kingdoms, *Basidiomycota* and *Ascomycota*, are the dominant phyla with the latter comprising over 1.5 million species. *Basidiomycota* is distinguished from *Ascomycota* by the presence of specialized cellular structures called basidia, where haploid sexual spores (basidiospores) are formed (Schmidt-Dannert, 2016). Despite the increasing proportions of P.SMS, a significant increase in *Basidiomycota* abundance was observed (*p* < 0.05). However, at the genus level, *Phanerochaete* exhibited consistent changes in abundance alongside *Basidiomycota*. This suggests that the observed variations in *Basidiomycota* are more likely attributable to fluctuations in *Phanerochaete* rather than to residuals of *Pleurotus ostreatus* in the diet. Furthermore, these results may also stem from the potential misidentification of *Pleurotus* as *Phanerochaete* during the amplicon analysis, possibly due to similarities in their genetic sequences targeted by the primers used. Additionally, we speculated that *Phanerochaete* may have also been present in the basal diet, which could explain its detection in the CON group. According to Datsomor et al. (2022), the co-culture of *P. chrysosporium* and *P. ostreatus* did not demonstrate a significant advantage over axenic cultures in achieving a substantial reduction in lignin content or enriching the biomass with holo-cellulose. This finding indicates a prevailing mutual intermingling and inhibitory relationship between *P. chrysosporium* and *P. ostreatus*, despite their ability to grow together on the same medium. The inhibition relationship between *P. chrysosporium* and *P. ostreatus* is likely responsible for the changes in *Basidiomycota* abundance.

In this experiment, a negative correlation was observed between *Prevotellaceae_UCG_001* (belonging to *Bacteroidota*) and *Cladosporium* (belonging to *Ascomycota*) among bacteria and fungi. *Prevotellaceae_UCG_001*, a strain of *Prevotella*, plays a crucial role in the degradation of various substrates, including hemicellulose, proteins, starches, xylan, and pectin (Zhang H. et al., 2022). This strain exhibited a strong correlation with the enrichment score of the AMPK signaling pathway, which is recognized as a vital sensor in regulating glycolipid metabolism (Song et al., 2019). *Cladosporium* is primarily known as a ubiquitous environmental saprophytic fungus or plant endophyte (Bensch et al., 2015). Although a few species have been documented as etiologic agents in vertebrate hosts, the number of such species identified to date remains limited (Sandoval-Denis et al., 2016). A significant positive correlation was found between *Cladosporium* abundance and isobutyrate concentration, while a significant negative correlation was observed between *Cladosporium* abundance and the levels of *β*-alanine and xanthine (Cui X. et al., 2022). *Ascomycota*, the most abundant fungal phylum across all groups in this study, comprises species that predominantly produce mycelia, which may contribute to the breakdown of plant cell walls (Wallace et al., 2019; Han et al., 2019). The pricise cause of this negative correlation remains unclear, necessitating further investigations to explore additional perspectives, such as examining the correlation between metabolites, to confirm and illuminate this relationship.

SMS shows promise as a feed resource due to its availability and potential to reduce the environmental impact of mushroom production residues. However, its nutritional consistency varies based on substrate types and cultivation practices, necessitating a thorough evaluation before integration into livestock diets. Processing methods aimed at enhancing digestibility and mitigating anti-nutritional factors are crucial considerations. Given that the substrate exhibits lower NDF and ADF after mushroom treatment, it can provide more fiber to microorganisms. However, the reduced carbohydrate content may limit its role in protein synthesis by these microorganisms. Therefore, proper formulation and feeding strategies are essential to realizing the benefits of P.SMS in livestock nutrition and sustainable farming practices.

## 5 Conclusion

In this experiment, we aimed to utilize P.SMS to determine the appropriate dosage and evaluate its effects on the rumen flora of Hu sheep. Alongside growth performance, rumen indices, and blood indices, our findings indicate that feeding a high percentage of P.SMS over an extended period adversely affects feed utilization efficiency, blood oxygen-carrying capacity, and kidney function, likely due to the influence of cottonseed hulls used in this study. Conversely, a moderate percentage of P.SMS appears to enhance rumen absorption capacity. Consistent with established expectations regarding rumen microbes, the addition of P.SMS impacted individual phylum-level and genus-level microorganisms; however, there is no evident rationale to implicate mushroom polysaccharides or mycelium. Furthermore, within the parameters of this experiment, no correlation was observed between changes in the microbial community structures of bacteria and fungi. In conclusion, substituting 10% of P.SMS for whole-plant corn silage proved to be the most effective approach in this study, demonstrating the feasibility of P.SMS feeding. However, the addition ratio is constrained by the P.SMS substrate, which varies by region. Notably, the substrate treated with white-rot fungi exhibited an enhancement in digestion and absorption in Hu sheep. Further experimental studies are needed to determine whether mushroom polysaccharides or mycelium affect rumen microbes.

## Data Availability

The datasets presented in this study can be found in online repositories. The names of the repository/repositories and accession number(s) can be found at: https://www.ncbi.nlm.nih.gov/, PRJNA1085887.

## Footnotes

^1^ github.com

^2^ drive5.com
