# Supplementary material for: Effects of spent substrate of oyster mushroom (Pleurotus ostreatus) on ruminal fermentation, microbial community and growth performance in Hu sheep
Source: Front Microbiol. 2024 Oct 23;15:1425218. doi: 10.3389/fmicb.2024.1425218 (PMC11538048; doi:10.3389/fmicb.2024.1425218)
Supplement: Supplementary file 1 [file Data_Sheet_1.docx]

Supplementary Material

**Supplementary Table 1.** Statistical Analysis of Sample Sequencing Data Processing Results

| Sample ID | 16S^*^ | | | | |  | ITS^*^ | | | | |
| --- | --- | --- | --- | --- | --- | --- | --- | --- | --- | --- | --- |
|  | Raw CCS | Clean CCS | Effective CCS | Average length(bp) | Effective(%) |  | Raw CCS | Clean CCS | Effective CCS | Average length(bp) | Effective(%) |
| CON1 | 7,054 | 6,972 | 6,867 | 1,456 | 97.35 |  | 7,631 | 7,630 | 7,611 | 614 | 99.74 |
| CON2 | 6,841 | 6,752 | 6,718 | 1,454 | 98.20 |  | 4,082 | 4,078 | 4,074 | 615 | 99.80 |
| CON3 | 7,477 | 7,404 | 7,369 | 1,455 | 98.56 |  | 7,582 | 7,582 | 7,579 | 649 | 99.96 |
| CON4 | 8,621 | 8,451 | 8,383 | 1,457 | 97.24 |  | 7,418 | 7,416 | 7,402 | 619 | 99.78 |
| CON5 | 6,951 | 6,713 | 6,704 | 1,457 | 96.45 |  | 7,463 | 7,462 | 7,445 | 615 | 99.76 |
| PSMS5-1 | 6,887 | 6,737 | 6,601 | 1,457 | 95.85 |  | 7,459 | 7,455 | 7,429 | 593 | 99.60 |
| PSMS5-2 | 8,169 | 7,858 | 7,849 | 1,457 | 96.08 |  | 7,067 | 7,065 | 7,035 | 618 | 99.55 |
| PSMS5-3 | 6,800 | 6,784 | 6,682 | 1,458 | 98.26 |  | 7,651 | 7,651 | 7,647 | 636 | 99.95 |
| PSMS5-4 | 8,183 | 7,907 | 7,879 | 1,458 | 96.28 |  | 7,226 | 7,225 | 7,181 | 621 | 99.38 |
| PSMS5-5 | 6,820 | 6,734 | 6,719 | 1,458 | 98.52 |  | 6,564 | 6,560 | 6,537 | 601 | 99.59 |
| PSMS10-1 | 6,968 | 6,784 | 6,729 | 1,458 | 96.57 |  | 7,833 | 7,831 | 7,800 | 597 | 99.58 |
| PSMS10-2 | 7,928 | 7,761 | 7,741 | 1,456 | 97.64 |  | 7,154 | 7,154 | 7,129 | 640 | 99.65 |
| PSMS10-3 | 8,268 | 8,144 | 7,886 | 1,455 | 95.38 |  | 7,856 | 7,856 | 7,840 | 646 | 99.80 |
| PSMS10-4 | 7,578 | 7,443 | 7,431 | 1,459 | 98.06 |  | 8,308 | 8,307 | 8,285 | 636 | 99.72 |
| PSMS10-5 | 7,369 | 7,356 | 7,294 | 1,458 | 98.98 |  | 7,090 | 7,090 | 7,069 | 623 | 99.70 |
| PSMS15-1 | 7,380 | 7,290 | 7,011 | 1,455 | 95.00 |  | 7,514 | 7,512 | 7,489 | 628 | 99.67 |
| PSMS15-2 | 7,200 | 6,947 | 6,941 | 1,459 | 96.40 |  | 7,558 | 7,556 | 7,492 | 640 | 99.13 |
| PSMS15-3 | 8,481 | 8,149 | 8,135 | 1,457 | 95.92 |  | 7,339 | 7,337 | 7,319 | 618 | 99.73 |
| PSMS15-4 | 8,048 | 7,884 | 7,849 | 1,457 | 97.53 |  | 8,417 | 8,417 | 8,363 | 642 | 99.36 |
| PSMS15-5 | 7,014 | 6,841 | 6,799 | 1,455 | 96.93 |  | 5,211 | 5,209 | 5,177 | 614 | 99.35 |
| PSMS20-1 | 8,073 | 7,804 | 7,796 | 1,458 | 96.57 |  | 8,098 | 8,096 | 8,054 | 653 | 99.46 |
| PSMS20-2 | 7,133 | 6,865 | 6,836 | 1,457 | 95.84 |  | 7,852 | 7,850 | 7,808 | 624 | 99.44 |
| PSMS20-3 | 7,229 | 7,169 | 7,108 | 1,457 | 98.33 |  | 7,419 | 7,418 | 7,400 | 643 | 99.74 |
| PSMS20-4 | 7,639 | 7,258 | 7,245 | 1,457 | 94.84 |  | 7,426 | 7,425 | 7,401 | 637 | 99.66 |
| PSMS20-5 | 7,206 | 6,964 | 6,951 | 1,457 | 96.46 |  | 7,554 | 7,554 | 7,529 | 622 | 99.67 |

**^*^** Raw-CCS: Counts of identified CCS reads in the sample; Clean CCS: Counts of clean CCS reads (post primer removal and length filtration); Effective-CCS: Counts of effective CCS reads after chimeric reads removal; Average length(bp): Average length reads in the sample; Effective(%): Percentage of effective CCS reads in raw reads.

**Supplementary Table 2.** Biological classification of bacterial and fungal samples

| Sample ID | 16S | | | | | | |  | ITS | | | | | | |
| --- | --- | --- | --- | --- | --- | --- | --- | --- | --- | --- | --- | --- | --- | --- | --- |
|  | Kindom | Phylum | Class | Order | Family | Genus | Species |  | Kindom | Phylum | Class | Order | Family | Genus | Species |
| CON1 | 1 | 7 | 16 | 24 | 36 | 47 | 58 |  | 1 | 12 | 18 | 38 | 60 | 94 | 116 |
| CON2 | 1 | 4 | 12 | 16 | 26 | 31 | 41 |  | 1 | 13 | 20 | 36 | 55 | 76 | 81 |
| CON3 | 1 | 7 | 14 | 20 | 24 | 34 | 48 |  | 1 | 10 | 16 | 26 | 41 | 53 | 60 |
| CON4 | 1 | 9 | 13 | 21 | 39 | 52 | 68 |  | 1 | 12 | 21 | 33 | 54 | 85 | 90 |
| CON5 | 1 | 7 | 14 | 23 | 35 | 44 | 61 |  | 1 | 11 | 19 | 33 | 48 | 69 | 83 |
| PSMS5-1 | 1 | 8 | 17 | 23 | 34 | 43 | 55 |  | 1 | 14 | 21 | 32 | 52 | 85 | 98 |
| PSMS5-2 | 1 | 8 | 17 | 27 | 43 | 56 | 71 |  | 1 | 8 | 14 | 27 | 43 | 49 | 56 |
| PSMS5-3 | 1 | 6 | 16 | 24 | 34 | 47 | 57 |  | 1 | 12 | 18 | 33 | 54 | 80 | 93 |
| PSMS5-4 | 1 | 7 | 18 | 25 | 38 | 47 | 61 |  | 1 | 12 | 19 | 32 | 50 | 68 | 83 |
| PSMS5-5 | 1 | 7 | 14 | 18 | 28 | 36 | 46 |  | 1 | 11 | 16 | 27 | 44 | 67 | 77 |
| PSMS10-1 | 1 | 6 | 13 | 19 | 30 | 41 | 60 |  | 1 | 11 | 16 | 26 | 49 | 78 | 92 |
| PSMS10-2 | 1 | 6 | 14 | 25 | 36 | 47 | 59 |  | 1 | 15 | 22 | 35 | 56 | 88 | 98 |
| PSMS10-3 | 1 | 8 | 17 | 26 | 37 | 48 | 65 |  | 1 | 13 | 20 | 35 | 51 | 81 | 91 |
| PSMS10-4 | 1 | 7 | 16 | 25 | 40 | 47 | 63 |  | 1 | 7 | 11 | 22 | 39 | 55 | 70 |
| PSMS10-5 | 1 | 6 | 15 | 21 | 29 | 40 | 47 |  | 1 | 11 | 16 | 27 | 41 | 67 | 72 |
| PSMS15-1 | 1 | 8 | 17 | 27 | 40 | 51 | 64 |  | 1 | 14 | 23 | 42 | 70 | 106 | 126 |
| PSMS15-2 | 1 | 8 | 15 | 23 | 35 | 46 | 59 |  | 1 | 7 | 13 | 23 | 39 | 56 | 68 |
| PSMS15-3 | 1 | 5 | 15 | 26 | 39 | 51 | 66 |  | 1 | 11 | 18 | 30 | 41 | 60 | 71 |
| PSMS15-4 | 1 | 7 | 15 | 27 | 44 | 62 | 90 |  | 1 | 14 | 21 | 35 | 58 | 87 | 97 |
| PSMS15-5 | 1 | 6 | 15 | 20 | 32 | 47 | 63 |  | 1 | 13 | 22 | 37 | 54 | 83 | 99 |
| PSMS20-1 | 1 | 6 | 17 | 23 | 36 | 48 | 57 |  | 1 | 8 | 13 | 26 | 43 | 50 | 63 |
| PSMS20-2 | 1 | 7 | 15 | 24 | 37 | 55 | 70 |  | 1 | 8 | 13 | 22 | 42 | 63 | 75 |
| PSMS20-3 | 1 | 8 | 15 | 23 | 31 | 40 | 57 |  | 1 | 11 | 20 | 34 | 53 | 73 | 95 |
| PSMS20-4 | 1 | 7 | 14 | 21 | 37 | 52 | 62 |  | 1 | 9 | 15 | 29 | 39 | 50 | 58 |
| PSMS20-5 | 1 | 8 | 21 | 37 | 55 | 81 | 97 |  | 1 | 8 | 15 | 28 | 42 | 56 | 64 |
| Total | 1 | 10 | 30 | 55 | 109 | 183 | 273 |  | 1 | 15 | 25 | 54 | 85 | 135 | 165 |

**Supplementary Table 3.** Composition of rumen bacteria and fungus at the phylum level %

| Composition^*^ | Groups | | | | | *P*-value |
| --- | --- | --- | --- | --- | --- | --- |
|  | CON | PSMS5 | PSMS10 | PSMS15 | PSMS20 |  |
| **Bacteria** |  |  |  |  |  |  |
| *Firmicutes* | 30.61 | 41.84 | 40.85 | 34.76 | 28.39 | 0.309 |
| *Bacteroidota* | 32.49 | 30.44 | 26.78 | 34.77 | 34.88 | 0.864 |
| *Verrucomicrobiota* | 9.56 | 15.06 | 12.55 | 16.46 | 11.45 | 0.211 |
| *Actinobacteriota* | 15.1 | 5.3 | 7.85 | 7.38 | 6.99 | 0.663 |
| *Planctomycetota* | 7.8 | 2.91 | 8.08 | 1.82 | 8.78 | 0.257 |
| *Patescibacteria* | 2.72^a^ | 3.18^a^ | 2.86^a^ | 3.25^a^ | 1.37^b^ | 0.026 |
| *Proteobacteria* | 0.67 | 0.31 | 0.22 | 0.4 | 7.88 | 0.069 |
| *Cyanobacteria* | 0.63 | 0.27 | 0.35 | 0.45 | 0.08 | 0.056 |
| *Desulfobacterota* | 0.07 | 0.2 | 0.11 | 0.15 | 0.06 | 0.331 |
| *Bdellovibrionota* | 0.09 | 0.06 | 0.18 | 0.08 | 0.02 | 0.478 |
| *Others* | 0.14 | 0.28 | 0.15 | 0.45 | 0.03 | 0.212 |
| *Unassigned* | 0.12 | 0.15 | 0.02 | 0.03 | 0.07 | 0.544 |
| Total | 100 | 100 | 100 | 100 | 100 |  |
| **Fungus** |  |  |  |  |  |  |
| *Ascomycota* | 54.84 | 69.8 | 55.51 | 54.49 | 49.77 | 0.162 |
| *Neocallimastigomycota* | 34.16 | 11.1 | 30.11 | 14.92 | 25 | 0.154 |
| *Basidiomycota* | 10.21^c^ | 18.16^abc^ | 13.66^bc^ | 28.81^a^ | 24.51^ab^ | 0.014 |
| *Chytridiomycota* | 0.05 | 0.1 | 0.09 | 1.32 | 0.16 | 0.476 |
| *Rozellomycota* | 0.27 | 0.42 | 0.16 | 0.12 | 0.13 | 0.423 |
| *Mucoromycota* | 0.22 | 0.25 | 0.3 | 0.18 | 0.13 | 0.515 |
| *Mortierellomycota* | 0.07 | 0.12 | 0.13 | 0.12 | 0.19 | 0.829 |
| *unclassified_Fungi* | 0.16 | 0.02 | 0.04 | 0.04 | 0.06 | 0.513 |
| *Glomeromycota* | 0.01 | 0.03 | 0 | 0 | 0.05 | 0.608 |
| *Zoopagomycota* | 0.01 | 0 | 0 | 0 | 0 | 0.492 |
| Total | 100 | 100 | 100 | 100 | 100 |  |

*****In the same row, values with no letter or the same letter superscripts indicate no significant difference (P > 0.05), while values with different letter superscripts indicate a significant difference (P < 0.05). The same applies below.

**Supplementary Table 4.** Composition of rumen bacteria and fungus at the genus level %

| composition | Groups | | | | | *P*-value |
| --- | --- | --- | --- | --- | --- | --- |
|  | CON | PSMS5 | PSMS10 | PSMS15 | PSMS20 |  |
| **Bacteria** |  |  |  |  |  |  |
| *Prevotella* | 22.07 | 20.75 | 19.23 | 21.96 | 27.42 | 0.890 |
| *uncultured_rumen_bacterium* | 14.54 | 17.74 | 16.96 | 22.51 | 13.21 | 0.132 |
| *Christensenellaceae_R_7_group* | 10.56 | 13.97 | 14.87 | 11.44 | 11.27 | 0.844 |
| *NK4A214_group* | 7.75 | 12.67 | 8.61 | 9.22 | 6.47 | 0.241 |
| *unclassified_Bifidobacteriaceae* | 15.10 | 5.30 | 7.84 | 7.38 | 6.99 | 0.663 |
| *Pirellula* | 7.80 | 2.91 | 8.08 | 1.82 | 8.78 | 0.257 |
| *Rikenellaceae_RC9_gut_group* | 6.11 | 7.13 | 3.80 | 7.31 | 4.84 | 0.416 |
| *Candidatus_Saccharimonas* | 2.71^ab^ | 3.18^a^ | 2.86^a^ | 3.09^a^ | 1.35^b^ | 0.025 |
| *Pseudomonas* | 0.02 | 0.11 | 0.07 | 0.08 | 6.37 | 0.056 |
| *UCG_005* | 0.89 | 2.10 | 1.48 | 0.77 | 0.52 | 0.558 |
| *Others* | 12.35 | 13.99 | 16.18 | 14.38 | 12.71 | 0.817 |
| *Unassigned* | 0.12 | 0.15 | 0.02 | 0.03 | 0.07 | 0.544 |
| Total | 100 | 100 | 100 | 100 | 100 |  |
| **Fungus** |  |  |  |  |  |  |
| *Trichoderma* | 11.68 | 25.52 | 21.25 | 25.47 | 23.33 | 0.304 |
| *Aspergillus* | 21.67 | 27.32 | 24.35 | 15.70 | 18.17 | 0.645 |
| *Phanerochaete* | 7.78^c^ | 16.66^abc^ | 11.79^bc^ | 27.49^a^ | 23.14^ab^ | 0.012 |
| *Orpinomyces* | 19.78 | 3.14 | 17.97 | 3.47 | 5.94 | 0.061 |
| *Caecomyces* | 0.08 | 1.62 | 4.43 | 3.59 | 8.72 | 0.523 |
| *Anaeromyces* | 4.93 | 2.83 | 2.29 | 2.66 | 2.48 | 0.620 |
| *Cyllamyces* | 4.39 | 1.46 | 3.89 | 0.71 | 2.52 | 0.391 |
| *Pichia* | 1.23 | 7.03 | 0.56 | 1.96 | 0.24 | 0.535 |
| *unclassified_Neocallimastigaceae* | 0.12 | 1.62 | 1.39 | 1.46 | 4.48 | 0.232 |
| *unidentified* | 2.43 | 1.53 | 0.81 | 3.56 | 0.72 | 0.162 |
| *Others* | 25.92 | 11.26 | 11.28 | 13.93 | 10.25 | 0.005 |
| Total | 100 | 100 | 100 | 100 | 100 |  |

**
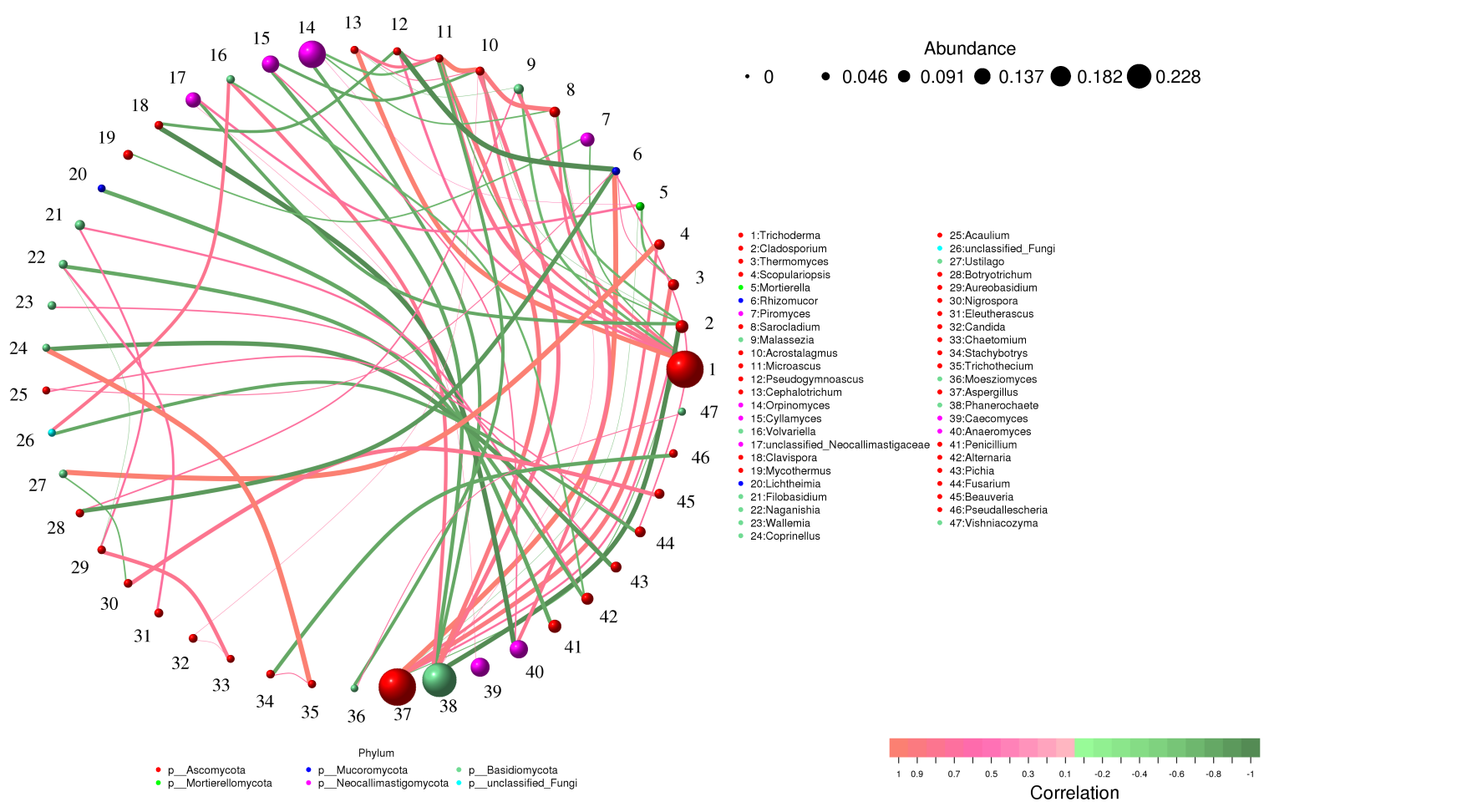
**

**Supplementary Figure 1.** Correlation network of top 50 genera for rumen fungi.Species are represented by circles, where the size of each circle indicates the abundance of the species. Furthermore, different colors are used to denote distinct phylum classifications. Edges represent the correlation between two species, with line thickness indicating strength and line color indicating correlation direction: orange for positive correlation and green for negative correlation.
